# Centrifugal Microfluidic Synthesis of Nickel Sesquioxide Nanoparticles

**DOI:** 10.3390/mi14091741

**Published:** 2023-09-06

**Authors:** Jiayou Mou, Chenxi Wang, Hongyi Zhao, Chuwei Xiong, Yong Ren, Jing Wang, Dan Jiang, Zansheng Zheng

**Affiliations:** 1 Research Group for Fluids and Thermal Engineering, University of Nottingham Ningbo China, Ningbo 315100, China; jiayou.mou@nottingham.edu.cn; 2 Department of Mechanical, Materials and Manufacturing Engineering, University of Nottingham Ningbo China, Ningbo 315100, China; shyhz5@nottingham.edu.cn (H.Z.); ssycx1@nottingham.edu.cn (C.X.); 3 Chemical and Environmental Engineering, University of Nottingham Ningbo China, Ningbo 315100, China; chenxi.wang@nottingham.edu.cn; 4 New Materials Institute, University of Nottingham Ningbo China, Ningbo 315100, China; 5 Key Laboratory of Carbonaceous Wastes Processing and Process Intensification Research of Zhejiang Province, University of Nottingham Ningbo China, Ningbo 315100, China; 6 Nottingham Ningbo China Beacons of Excellence Research and Innovation Institute, University of Nottingham Ningbo China, Ningbo 315154, China; 7 Department of Electrical and Electronic Engineering, University of Nottingham Ningbo China, Ningbo 315100, China; 8 Key Laboratory of More Electric Aircraft Technology of Zhejiang Province, University of Nottingham Ningbo China, Ningbo 315100, China; 9 Ningbo Chemgoo Pharma Tech Co., Ltd., Ningbo 315800, China; jiangdan@chemgoo.com

**Keywords:** centrifugal microfluidic, nickel sesquioxide, nanoparticles

## Abstract

Nickel sesquioxide (Ni_2_O_3_) nanoparticles were synthesized using centrifugal microfluidics in the present study. The obtained nanoparticles were characterized using SEM to investigate their morphology and microstructure, and XRD was employed to analyze their purity. The nanoparticle size data were measured and analyzed using ImageJ (v1.8.0) software. The flow process and mixing procedure were monitored through computational fluid dynamics simulation. Among the synthesized Ni_2_O_3_ nanoparticles, those obtained at the rotation speed of 1000 rpm for 10 min with angular acceleration of 4.2 rad/s^2^ showed the best performance in terms of high purity, complete shape and microstructure, small diameter, and narrow diameter distribution. The experimental results demonstrate that the rotation speed of the microfluidic chip and reaction time contribute to a decrease in particle diameter and a narrower diameter distribution range. In contrast, an increase in acceleration of the rotation speed leads to an expanded nanoparticle size range and, thus, a wider distribution. These findings contribute to a comprehensive understanding of the effects exerted by various factors in centrifugal microfluidics and will provide new insights into nanoparticle synthesis using centrifugal microfluidic technology.

## 1. Introduction

The synthesis of nanoparticles with tailored properties has garnered significant attention in scientific and technological research, driven by their unique characteristics and versatile applications. Nickel oxide (Ni_2_O_3_) nanoparticles, in particular, have emerged as promising materials due to their remarkable electrical, optical, and magnetic properties [1,2,3]. However, conventional synthesis methods often encounter challenges in achieving precise control over nanoparticle size, shape, and composition, limiting their potential for practical applications [4,5]. To overcome these limitations, microfluidic technology has emerged as a powerful approach for the synthesis of Ni_2_O_3_ nanoparticles, offering enhanced control, reproducibility, and scalability.

Microfluidics, which involves the manipulation of fluids at the microscale, has gained prominence in recent years owing to its ability to precisely control reaction conditions and exhibit unique fluidic behaviors [6,7,8]. Microfluidic platforms provide numerous advantages for nanoparticle synthesis, including superior mixing efficiency [9], rapid heat transfer [7], and the capability to perform multiple steps in a controlled manner [10]. Wu et al. [11] introduced a current-flow field in micro-mixer, which significantly improved the mixing efficiency. Sasaki et al. [12] similarly introduced a pair of coplanar electrodes to enhance the mixing efficiency. These features enable precise control over the nucleation, growth, and assembly processes, facilitating the synthesis of Ni_2_O_3_ nanoparticles with tailored properties.

Several synthesis strategies have been successfully implemented in microfluidic systems for the controlled fabrication of nanoparticles [7,13]. For instance, Lian et al. effectively connected microfluidics in manufacturing TiO_2_ nanoparticles through a surfactant wrapping sol-gel strategy, with TiO_2_ nanoparticles equitably conveyed on the external surface of the multi-wall carbon nanotubes (MWCNTs), and it has the capacity to photodegrade Rhodamine B for wastewater treatment [14].

Characterization of the synthesized Ni_2_O_3_ nanoparticles is critical for understanding their structural, morphological, and compositional properties [15,16,17,18]. Techniques such as X-ray diffraction (XRD), transmission electron microscopy (TEM), scanning electron microscopy (SEM), and Fourier-transform infrared spectroscopy (FTIR) can provide valuable insights into the crystal structure, size, shape, and surface chemistry. These characterization techniques enable researchers to establish correlations between nanoparticle properties and their performance in specific applications, facilitating further optimization and customization.

Therefore, microfluidic synthesis offers a promising pathway for the controlled fabrication of Ni_2_O_3_ nanoparticles with enhanced properties. The precise control over reaction conditions, uniform size distribution, and tunable morphology and crystallinity of the nanoparticles open up exciting possibilities for their integration into various energy and environmental applications. Among various microfluidic systems, centrifugal microfluidics has demonstrated superior properties in comparison to the conventional pump-driven microchips. For instance, it requires only a compact motor to generate the centrifugal pumping for fluid manipulation and, hence, will eliminate the need for syringe pumps. The complete fluidic network can be contained in a single disc. The different fluidic functions such as valving, decanting, calibration, and mixing, as well as sample splitting and separation, have been successfully integrated on the centrifugal microfluidic platform. Furthermore, the centrifugal microfluidic disk can be fabricated to be disposable in an economical way by mass production from inexpensive materials such as polycarbonate. To the best of our knowledge, there has been no work reporting the synthesis of Ni_2_O_3_ nanoparticles using centrifugal microfluidics. By exploring recent advancements in microfluidic synthesis, this research paper, therefore, aims to provide a new strategy using a centrifugal microfluidic platform for the synthesis of nanomaterials.

## 2. Simulation Setting

The simulation was conducted using Ansys Fluent to investigate the flow and mixing performance of two different fluids in a microchannel patterned in a rotating microchip. The flow conditions were analyzed in terms of velocity vectors and streamlines. The simulation model employed the volume of fluid approach with energy and gravity enabled. The turbulent flow using the Shear Stress Transport (SST) k-omega viscous model was applied in the present study.

Two fluids, alcohol and water, were adopted as the testing samples in the simulation, with the water temperature set at 400 K and alcohol at 300 K. In the stationary flow condition, an inlet velocity of 2 m/s was assigned to the flow fluid. For the centrifugal condition, a rotational speed of 500 rpm was applied. To analyze the impact of the flow induced by the centrifugal microfluidic platform, the velocity vector distribution and phase distribution in these two conditions were compared.

On a rotating microfluidic chip, the fluid in the microchannel will experience the centrifugal force as shown in Equation (1),

(1) $$F_{c}=\rho r\omega^{2}$$

the Euler force for non-uniform velocity condition in Equation (2),

(2) $$F_{E}=\rho r\times d\omega /dt$$

and the Coriolis force in Equation (3).

(3) $$F_{cr}=2\rho \omega v$$

The Navier–Stokes equations governing the conservation of momentum in a viscous fluid are shown in Equation (4).

(4) $$\frac{\partial V}{\partial t}+\left(V\times \nabla \right)V=f-\frac{1}{\rho }\nabla p+\frac{\mu }{\rho }\nabla^{2}V$$

**Figure 1:**
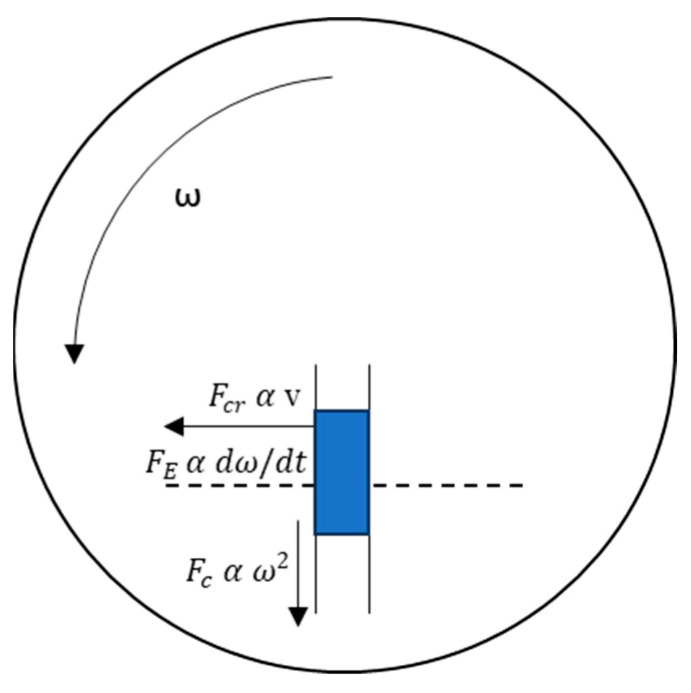
Schematic of the forces working in fluids flowing through a microchannel located in a rotating centrifugal microfluidic chip.

In the above equations, ρ represents density, *r* is radius, ω is angular velocity, *t* is time, *v* is velocity, *V* is velocity vector, *p* is pressure, *f* is the external force per unit volume of fluid, if only gravity is considered, then *f* = ρ*g*, *g* is gravity, μ is dynamic viscosity. Figure 1 depicts the governing forces exerting influence on the fluids in the rotating microchannel.

**Figure 2:**
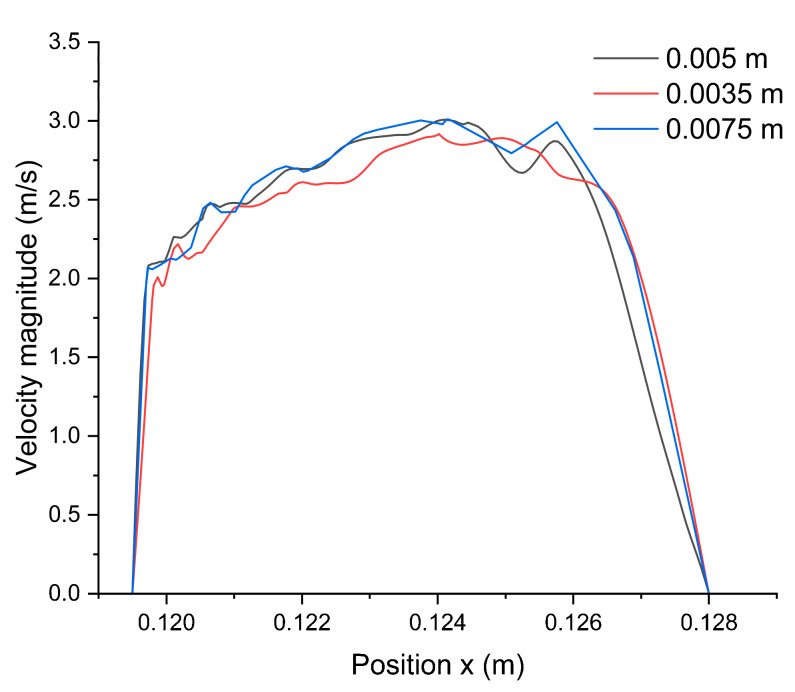
Mesh-sensitive analyzed results.

**Figure 3:**
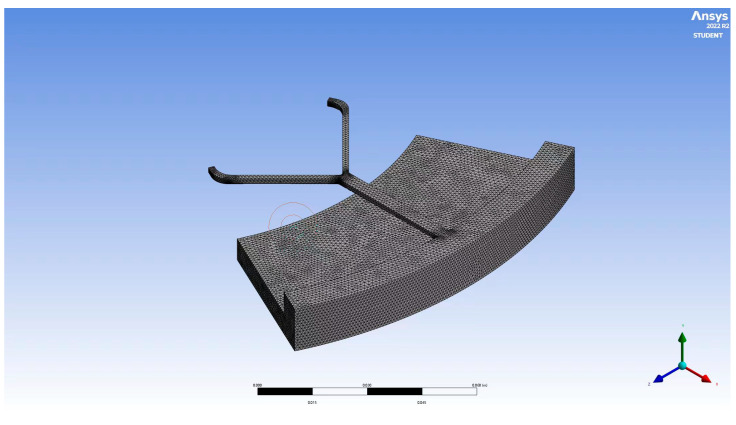
The created mesh of microchannel in the centrifugal microfluidic chip.

A mesh-size-sensitive study was conducted to investigate the reliability of the numerical study. The simulation was run under different mesh sizes, 0.005, 0.0035, and 0.0075 m, respectively, to compare the corresponding velocity magnitude to validate the numerical accuracy. As shown in Figure 2, the plot of results using mesh sizes of 0.005 and 0.0075 m are similar, and the results of 0.0035 m are relatively close to the results of 0.005 m; therefore, the mesh element size of 0.005 m was applied in the following simulation, which is shown in Figure 3.

## 3. Material and Experiment Method

**Figure 4:**
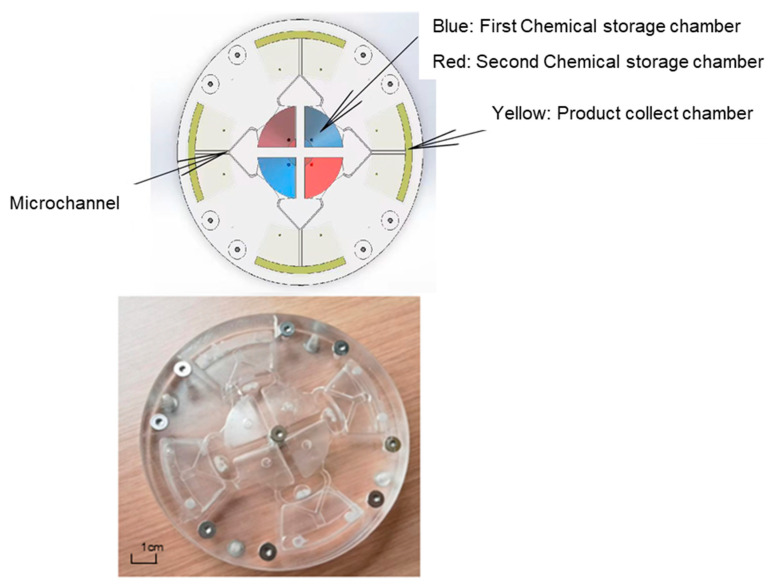
The model (**top**) and photo (**bottom**) of the centrifugal microfluidic chip.

All chemicals were purchased from Aladdin Company, and the chemicals were directly used without any purification. For a typical experiment, 0.8 g Ni(NO_3_)_2_∙6H_2_O was dissolved in 15 mL deionized water, and was marked as solution A. A weight of 1.6 g NaOH was dissolved in 20 mL NaClO solution with 5% active chlorine in another beaker, and marked as solution B. The microfluidic chip is divided into three layers and made of acrylic, and three different molds were used, respectively, for fabrication. Hot pressing was applied for the three layers of the structure, which were then fixed together with screws to prevent leakage. The product is shown in Figure 4.The centrifugal microfluidic chip was fixed on a rotating machine. The solutions A and B were injected into the opposite 2 chambers, each corresponding to the red chamber and the blue chamber, respectively. The microfluidic chip can be reused several times; the design of the lower plate could help to clean, collect, and remove the waste inside the microfluidic chip, and the microfluidic chip could be easily cleaned and reused.

**Figure 5:**
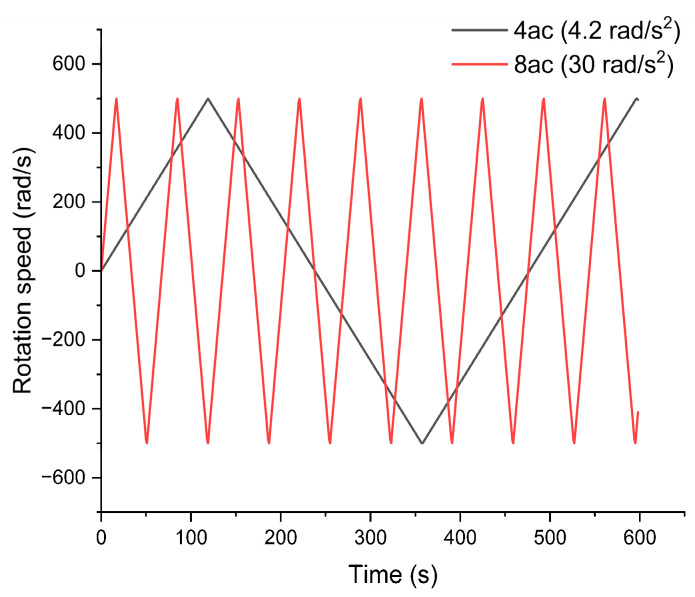
Cyclic rotational speed of microfluidic chip.

As shown in Figure 5, a programmed scheme was adopted to control the motor speed of the rotational platform, and a cyclic rotational speed with linear acceleration followed by linear deceleration was applied on the microfluidic chip, where 4-class acceleration (4ac) adopted the rotational acceleration/deceleration of ±4.2 rad/s^2^, and 8-class acceleration (8ac) adopted the rotational acceleration/deceleration of ±30 rad/s^2^.

Upon the start of the rotating machine, the two solutions quickly formed black particles, which were collected in the lower chamber. After rotation for 5 min, the machine was switched off, and the microfluidic chip was opened to collect the precipitate. The produced black precipitate (Ni_2_O_3_·xH_2_O) was further washed several times by a NaClO solution and deionized water. After filtration, the final dark black precipitate was placed in a 90-degree dryer for 12 h. Note that the temperature of the hot treatment cannot be too high; otherwise, the Ni_2_O_3_ will be reduced to NiO. Subsequently, the aggregate was ground to obtain the Ni_2_O_3_ powder in the form of nanoparticles. Compared to the conventional synthesis method, due to the rapid mixing generated from the centrifugal microfluidic platform, the chemical reactions were sped up in a pronounced way, which reduces the reaction procedure from hours to several minutes. With the conventional-method mixing, based on a large amount of solution with one-way rotation on a magnet mixer. For microfluidic synthesis, the amount of reactants in the chamber has been limited to a very low volume, with clockwise and anti-clockwise rotation. These factors significantly improve the mixing efficiency and reaction speed. However, the production rate is relatively low, as it is restricted by the size of microfluidic chip and chamber. The chemical reaction during the synthesis process is listed below in Equation (5).

(5) $${2(Ni({NO}_{3})}_{2}\cdot 6H_{2}0)+NaClO+4NaOH\to NaCl+{Ni}_{2}O_{3}↓+4Na({NO}_{3})+14H_{2}O$$

The precursors of nickel Ni(NO_3_)_2_∙6H_2_O dissolved in water will create Ni^2+^ and NO_3_^−^. After that, the sodium hypochlorite with 5% active chlorine will dissociate into Na^+^ and ClO^−^, ClO^−^ will be subject to self-decomposition into Cl^−^ and O_2_. However, the self-decomposition of ClO^−^ will be restrained in an alkaline condition, where more active ClO^−^ exists in the solution. Then ClO^−^ will react with Ni^2+^, the Cl^+^ ion in ClO^−^ will be reduced to Cl^−^; meanwhile, Ni^2+^ will lose an electron and be oxidized into Ni^3+^. The product of the solution reaction is Ni_2_O_3_·xH_2_O, which will be further washed with NaClO to guarantee the ion transformation from Ni^2+^ to Ni^3+^, and then washed several times with deionized water to remove the waste reaction product (i.e., NaCl and NaOH) as well as the 6xceedance amount of NaClO. Subsequently, the 90-degree dryer was used in the final step for H_2_O desorption in the black participate.

## 4. Characterization

The size, morphology, and microstructure of as-fabricated nanoparticles were investigated by a Field Emission scanning electron microscope (Zeiss SIGMA VP, Oberkochen, Germany). Crystal structure and purity of samples were examined by the X-ray diffraction (BRUKER D8 ADVANCE DAVINCI, Karlsruhe, Germany) Cu Kα radiation, λ = 1.5416 Å. The nanoparticle diameter data were analyzed by ImageJ.

## 5. Results and Discussion

### 5.1. Simulation Results

**Figure 6:**
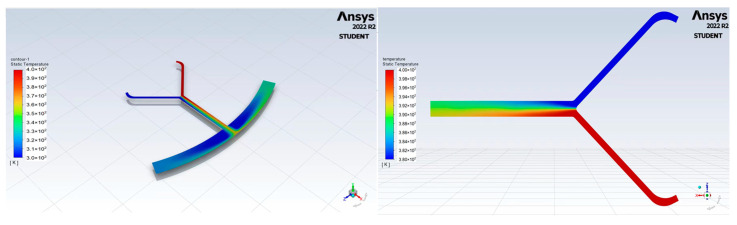
Plot of stationary condition of fluid flow in microchannel.

As depicted in Figure 6, when the microchannel is stationary, two fluids are introduced into the inlets of the microchannel, respectively. Initially, the two fluids are separated and maintained at different temperatures. Subsequently, the fluids flow into the microchannel downstream, leading to mixing between two phases. Minimal phase exchange is observed until the fluids reach the collection chamber. In summary, the two fluids exhibit relative stability with limited intermixing.

**Figure 7:**
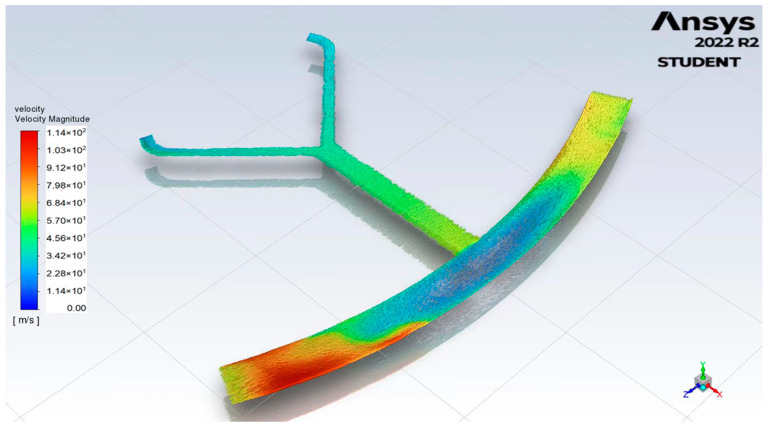
Plot of flow in centrifugal microfluidic channel.

Figure 7 displays the velocity vector distribution results of the microchannel, which is subject to a rotational scheme with a maximum speed of 500 rpm in a counterclockwise, followed by a clockwise, rotational manner. Evidently, the flow field becomes more unstable, and the interphase exchange significantly increases. Within the microchannel, the high-velocity region is observed at the distal portion of the mixing channels. In the collecting chamber, high velocities accumulate at both the left and right ends of the chamber. This phenomenon is attributed to the enhanced mixing due to the Coriolis-force-induced secondary flow in the microchannel [19,20], as well as the three-dimensional vortices in the collecting chamber [21].

### 5.2. Experiment Results

**Figure 8:**
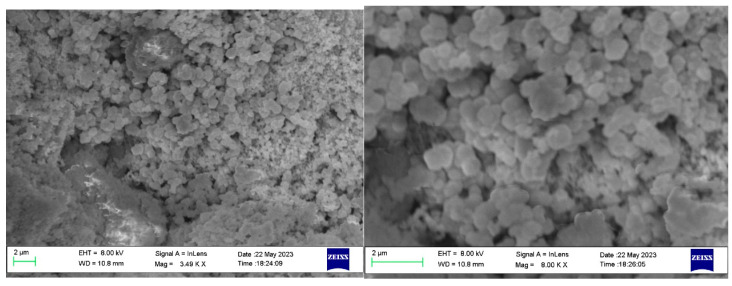
SEM results of synthesized Ni_2_O_3_ nanoparticles.

**Figure 9:**
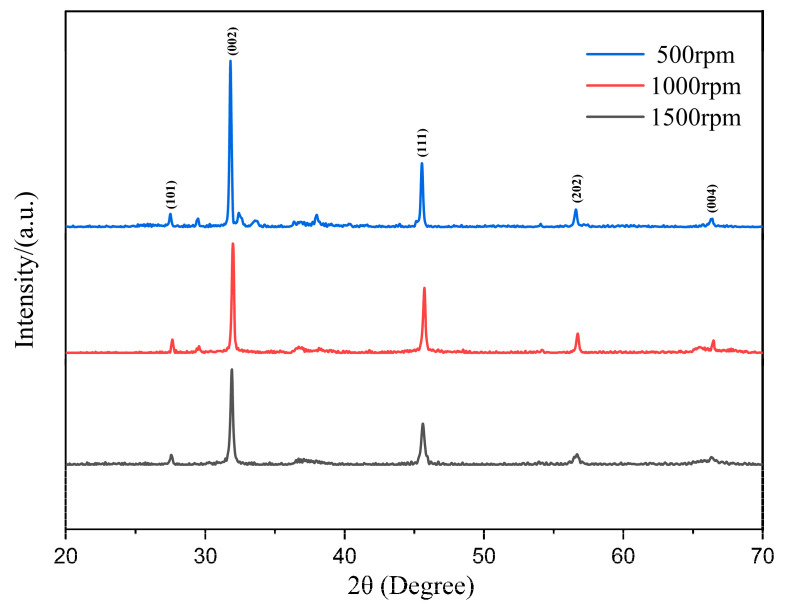
The X-ray diffraction pattern of synthesized Ni_2_O_3_ nanoparticles.

The morphology and microstructure of the as-synthesized nanoparticles were characterized using SEM, and the crystal structure and purity were investigated through XRD analysis, as shown in Figure 8 and Figure 9, respectively. The SEM results reveal the complete particle shape of Ni_2_O_3_, with the nanoparticles bonded together to form loose aggregates. Unlike the conventional synthesis methods that typically require several hours, the reaction time in this research was significantly reduced to 5 min. Furthermore, the study analyzed the effects of rotation speed and rotation acceleration on the particle diameter and purity. The particle diameters were calculated using ImageJ software, while the purity was assessed based on the XRD patterns.

**Figure 10:**
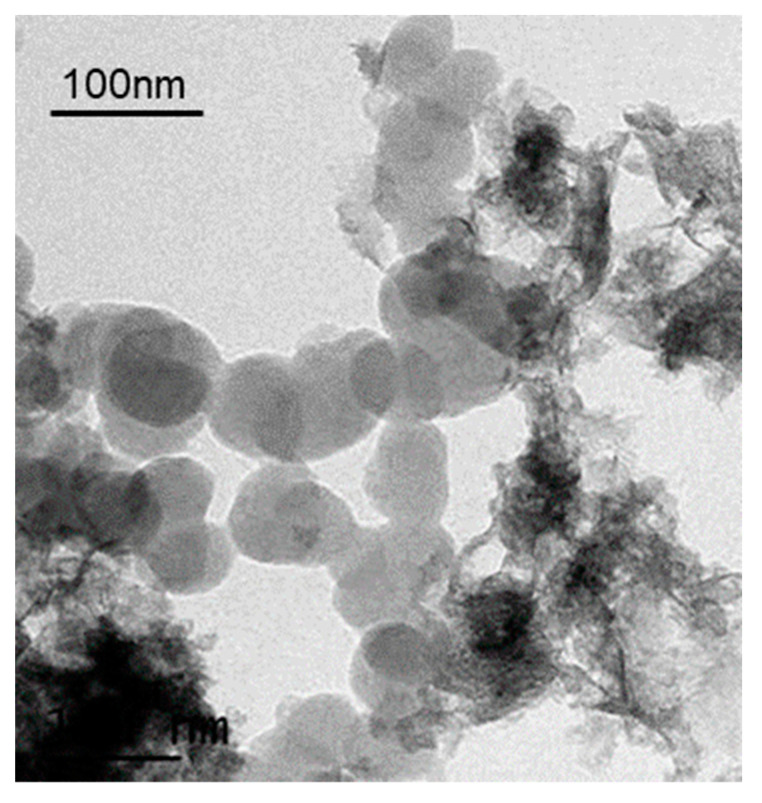
TEM results of synthesized Ni_2_O_3_ nanoparticles.

Figure 9 presents the XRD pattern of the particles synthesized at rotation speeds of 500 rpm, 1000 rpm, and 1500 rpm. The observed peaks at 27.64°, 31.66°, 45.61°, 56.58°, and 66.2° correspond to the crystal surfaces of the (101), (002), (111), (202), and (004) planes, respectively. Figure 10 shows the TEM result of synthesized Ni_2_O_3_ nanoparticles; it can be summarized as that the nanoparticles have a relatively complete sphere shape, the size varies from 30 nm to 80 nm. There is also some impurity content shown in the figure; there should be a NaCl crystal and NaOH. This also explains the wave peaks in the XRD pattern.

The synthesized process involved a pure 5 min reaction without any purification or washing procedures. Consequently, the XRD results exhibit miscellaneous peaks due to the presence of impurities, primarily sodium chloride (NaCl), which is a byproduct of the reaction. The experiment needs an excess amount of sodium hydroxide; therefore, the final product may contain NaOH when washing is incomplete. Comparing the three rotation speeds, the 500 rpm group exhibits additional peaks around the main peak of Ni_2_O_3_ (31.66°), indicating relatively lower particle purity. As the rotation speed increases, the occurrence of miscellaneous peaks diminishes, and the curve around 31.66° becomes smoother. In the 1500 rpm group, the curve appears smooth with minimal disturbances, confirming the high purity of the crystal surface.

**Figure 11:**
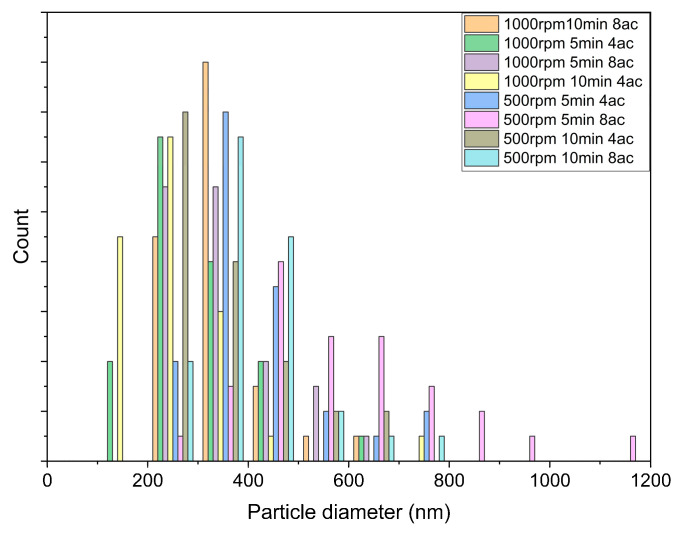
The particle diameter distribution of all groups.

The diameters of synthesized Ni_2_O_3_ nanoparticles are plotted in Figure 11. Following the statistical method, we used ImageJ to recognize the sphere-shaped particles in the SEM results, and randomly picked 40 particles from the SEM pictures, counted the diameter of the particles, and analyzed the distribution and curve. Moreover, the symbols 4ac and 8ac represent a rotational acceleration/deceleration of ±4.2 rad/s^2^, and ±30 rad/s^2^, respectively.

**Figure 12:**
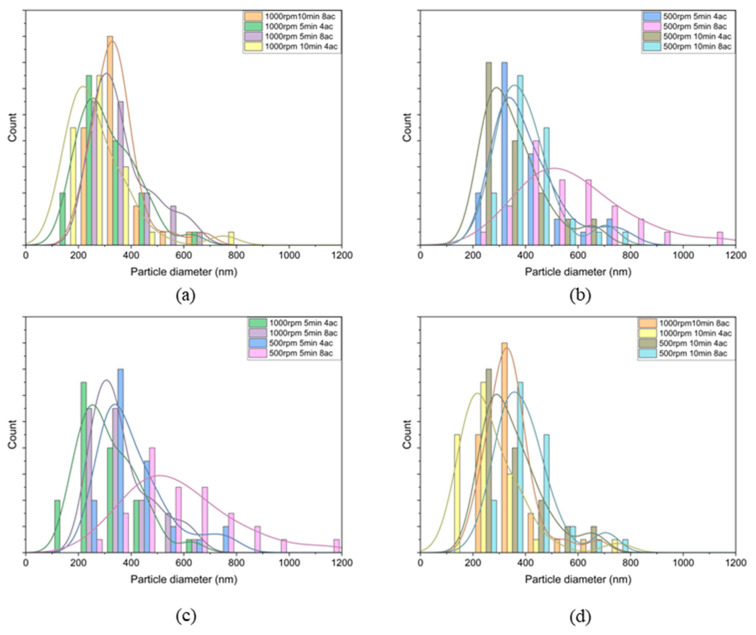
The diameter distribution of different groups (**a**) at 1000 rpm with different mixing times and accelerations; (**b**) at 500 rpm with different mixing times and accelerations; (**c**) at 5 min with different rotation speeds and accelerations; (**d**) at 10 min with different rotation speeds and accelerations.

To investigate the influence of different factors on microfluidic synthesis, the data were divided into four groups for analysis of their effects separately. Figure 12a illustrates the results for the 1000 rpm condition. In this condition, the smallest diameter range observed is 100–200 nm. Within this range, the 1000 rpm 10 min 4ac group exhibits the highest particle count. Comparing the 5 min 4ac and 10 min 4ac groups, it can be concluded that as the reaction time increases, the particle diameter range shifts from larger sizes to smaller sizes. Approximately 80% of the particles in the 1000 rpm 10 min 4ac group are below 300 nm, whereas only about 60% of the particles in the 5 min 4ac group fall within that range. Similar observations can be made for the 1000 rpm 5 min 8ac and 1000 rpm 10 min 8ac groups, wherein the diameter ranges are more concentrated below 400 nm. Notably, nearly 90% of the particles in the 10 min 8ac group are below 400 nm, and it also exhibits the highest particle count within that range, indicating a narrower distribution of particle diameters.

Figure 12b demonstrates a similar trend. In the 500 rpm condition, the highest particle count for the 10 min 4ac group falls between 200–300 nm. Comparing this with the 5 min 4ac group, a clear shift in the peak can be observed from 300–400 nm to 200–300 nm. This effect becomes more pronounced when comparing the 5 min 8ac and 10 min 8ac groups. The diameter distribution for 5 min 8ac is broader, with a mean particle diameter of over 580 nm. However, with an increase in reaction time, the diameter distribution becomes more concentrated and shifts towards a lower range.

**Figure 13:**
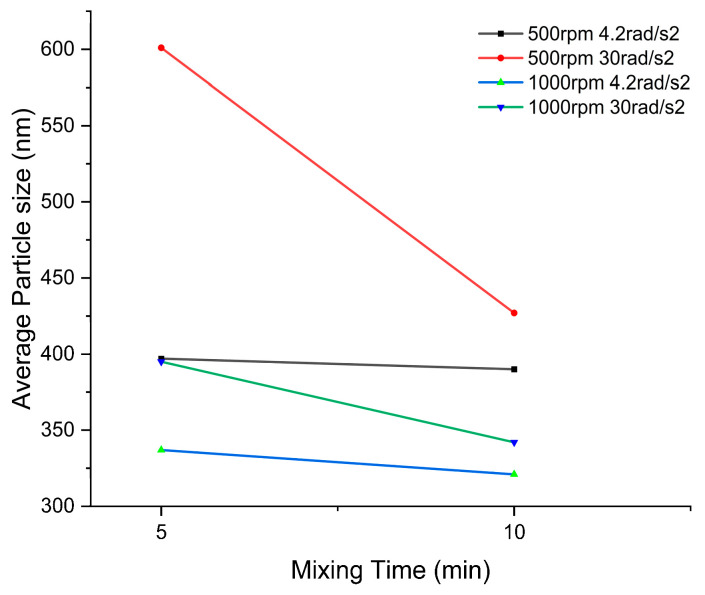
The effect of mixing time on the average of nanoparticle sizes.

The results from Figure 13 support these findings. With an increase in mixing time, the mean particle diameter significantly decreases for the 500 rpm 8ac and 1000 rpm 8ac groups. The effect is relatively smaller for the 500 rpm 4ac and 1000 rpm 4ac groups.

From Figure 12c,d, the effects of rotation speed are analyzed. At the same reaction time and acceleration, an increase in rotation speed leads to a decrease in particle diameter and a narrowing of the diameter distribution. In the 5 min 4ac group, the performance of 1000 rpm 5 min 4ac is superior to that of 500 rpm 5 min 4ac. Over 90% of the particles in the 1000 rpm 5 min 4ac group are smaller than 400 nm, with the majority focusing around 200 nm. Conversely, for the 500 rpm 5 min 4ac group, the diameter range is more concentrated between 300 nm and 500 nm. Similar observations can be made for the 500 rpm 5 min 8ac and 1000 rpm 5 min 8ac groups, as well as for the 500 rpm 10 min 4ac and 1000 rpm 10 min 4ac groups.

In contrast, the acceleration factor exhibits different behavior compared to the other two factors. In the 5 min group, increasing the acceleration from 4 to 8 (representing an increase from 4.2 rad/s^2^ to 30 rad/s^2^) resulted in an increase in particle diameter. Comparing the 500 rpm 5 min 4ac and 500 rpm 5 min 8ac groups, as well as the 1000 rpm 5 min 4ac and 1000 rpm 5 min 8ac groups, the diameter distribution range shifts to a larger range with the increase in acceleration. A similar pattern is observed in the 10 min groups, where the 8ac groups exhibit larger diameters and wider diameter distributions compared to the 4ac groups.

## 6. Conclusions

Our work investigates the effects of various factors on the morphology, purity, particle size, and particle size distribution in the synthesis of Ni_2_O_3_ nanoparticles using centrifugal microfluidics, which has significantly reduced the processing time, from hours to 5–10 min, in comparison to conventional procedures. Thereby, it will provide new insights for nanoparticle synthesis using centrifugal microfluidics. This work demonstrates that the rotational speed of the microfluidic disc and the reaction duration contribute as determining roles to particle size reduction and a narrower particle size distribution. However, the rotational acceleration exhibits an inverse relationship, whereby an increase in acceleration leads to an enlargement of particle size and a wider particle size distribution. Further work will be required to improve the purity of the Ni_2_O_3_ nanoparticles by introducing additional purification processes, via washing with NaClO and deionized water to remove the waste reaction products, such as NaCl and NaOH.

## Data Availability

The data that support the findings of this study are available from the corresponding author upon reasonable request.
